# Biotechnological Advances in Sanguinarine and Chelerythrine Production from Plume Poppy (*Macleaya cordata*): A Gene Editing Perspective

**DOI:** 10.3390/plants14172667

**Published:** 2025-08-26

**Authors:** Bilal A. Rather, Wujun Xu, Aadil Yousuf Tantray, Moksh Mahajan, Huapeng Sun, Hanqing Cong, Xuefei Jiang, M. Iqbal R. Khan, Fei Qiao

**Affiliations:** 1 Sanya Institute of Breeding and Multiplication/School of Tropical Agriculture and Forestry, Hainan University, Sanya 572024, China; saffibilal@gmail.com (B.A.R.); xuwujun422@gmail.com (W.X.); 2 National Key Laboratory for Tropical Crop Breeding, Tropical Crops Genetic Resources Institute, Chinese Academy of Tropical Agricultural Sciences, Sanya 572024, China; huapeng_sun@catas.cn (H.S.); hanqing.cong@catas.cn (H.C.); 3 Department of Botany, University of Kashmir, Srinagar 190006, India; tantrayadil313@gmail.com; 4 Department of Botany, Jamia Hamdard, New Delhi 110062, India; 5 Department of Plant Biotechnology, Korea University, Seoul 02841, Republic of Korea

**Keywords:** chelerythrine, CRISPR/Cas9, plume poppy, sanguinarine

## Abstract

Plume poppy (*Macleaya cordata*), an important member of the Papaveraceae family, is a substantial source of benzylisoquinoline alkaloids (BIAs) such as sanguinarine and chelerythrine. These compounds possess significant therapeutic potential, including anti-inflammatory, anticancer, and antimicrobial activities, along with various industrial applications. However, the yield of these compounds in native plants are minimal and highly variable due to certain ecological factors. Recent advances in transgenic technologies have opened a new avenue for enhancing the biosynthesis of BIAs and optimizing their delivery in plume poppy. This review consolidates recent strategies in gene editing and metabolic modulations aimed at improving alkaloid biosynthesis in plume poppy. It uniquely connects these tools with industrial and therapeutic demands, offering a roadmap for enhanced BIA production. The current review also provides new insights into the overcoming the current limitations, offering potential solutions for stable, high-yield production of BIAs in plume poppy for their therapeutic use.

## 1. Introduction

Benzylisoquinoline alkaloids (BIAs) are a diverse group of plant derived compounds primarily found in the Papaveraceae, Berberidaceae, and Ranunculaceae families [1]. BIAs are an important class of nitrogenous secondary metabolites (SMs), structurally characterized by a benzylisoquinoline skeleton with nearly 2500 identified structures [2]. BIAs have long been recognized for their therapeutic properties, forming the basis of numerous medicines used in modern pharmacology [3,4]. Key BIAs, including chelerythrine, sanguinarine, and protopines, have shown to possess potent biological activities, such as antimicrobial and anticancer activities and anti-inflammatory effects [5]. Furthermore, sanguinarine serves as an alternative to the antibiotic growth promoters in animal farming, while chelerythrine has multiple important therapeutic applications [6]. Overall, these BIAs play essential roles in the human wellness and medicine industry. Their significance in the medicinal field prioritizes methods to enhance their synthesis and delivery, predominantly using synthetic biology and transgenic approaches. In recent years, the demand for these BIAs in the market has grown consistently; however, their application is greatly limited by their low natural abundance in plants. This limitation necessitates alternative strategies for their production, especially using plant engineering and synthetic biology.

*Macleaya cordata*, known in Chinese as “Bo-luo-hui” and commonly referred to as plume poppy, is a rich source of BIAs, belongs to the family Papaveraceae, and is broadly studied for its traditional medicinal applications. The plant is valued for its richness in SMs, with approximately 300 identified, including 204 isoquinoline alkaloids [7]. Its considerable commercial value and potential have attracted the pharmaceutical industry’s interest in enhancing its ability to produce SMs. Notably, it has been known to produce high concentration of antimicrobial BIAs such as chelerythrine, protopine, and sanguinarine [7,8]. Given its non-narcotic profile, which is lacking in compounds like morphine and codeine [9], it presents a safer alternative for pharmaceutical exploitation. The therapeutic applications have been profoundly evaluated to validate its traditional applications and efficiency as a remedy in ethnomedicine [10]. It is particularly valued for its production of natural growth promoters and exhibits anti-inflammatory, insecticidal, antimicrobial, and anticancer effects [4,11]. The plant has been well mentioned in ancient Chinese texts such as “Ben-Cao-Shi-Yi,” from the early Tang Dynasty [4,9]. Over the past years, *Macleaya* species have garnered recognition for their applications in livestock management [4]. In Germany, plume poppy is cultivated for use as an additive in animal feed and for biogas production. Additionally, it is listed as a plant utilized in feed additives for livestock production, according to the European Food Safety Authority (EFSA) [11].

In recent years, numerous research groups worldwide have extensively studied the biosynthesis of BIAs in plume poppy [7,9]. However, the advent of “omics” approaches in recent years, including genomics, transcriptomics, proteomics, and metabolomics, has greatly advanced our understanding of BIA biosynthesis. Genomics and transcriptomics played a crucial role in identifying genes and regulatory elements involved in BIA biosynthesis, while proteomics and metabolomics identified the functional proteins and metabolites [9,11]. The complete genomic and transcriptomic analyses of plume poppy provide valuable insights that support the development of its derivatives and plant metabolites for medicinal purposes [9]. Despite the growing interest, the natural production of BIAs in plume poppy is limited by environmental factors, developmental stages, and growth conditions [12]. Conventional breeding and cultivation techniques have not been sufficient to meet the growing demand for these compounds in the pharmaceutical industry. However, despite extensive pharmacological interest, natural production of sanguinarine and chelerythrine remains inefficient and unstable [13]. Traditional cultivation and breeding approaches have failed to significantly boost yields; therefore, modern biotechnological interventions, particularly gene editing, are being investigated to overcome these challenges for modulating growth and enhancing SM production in plume poppy [7]. Recent advances have focused on CRISPR/Cas9 (clustered regularly interspaced short palindromic repeats/CRISPR-associated protein 9) optimization and the overexpression of various genes involved in SM biosynthesis [14]. In this context, transgenic approaches offer a promising solution to enhance BIA production by manipulating the plant’s metabolic pathways and improving the stability of these bioactive compounds.

Although many studies have explored the impact of different plant growth techniques on BIA production, a comprehensive review that synthesizes the existing knowledge in this field is still needed. Therefore, this review article aims to highlight recent advancements in transgenic technologies aimed at boosting sanguinarine and chelerythrine production in plume poppy using gene editing and molecular farming strategies. The current study also explores the challenges and potential solutions for scaling up their production to meet the industrial demands.

## 2. Botanical Description of *Macleaya cordata* (Plume Poppy)

**Figure 1:**
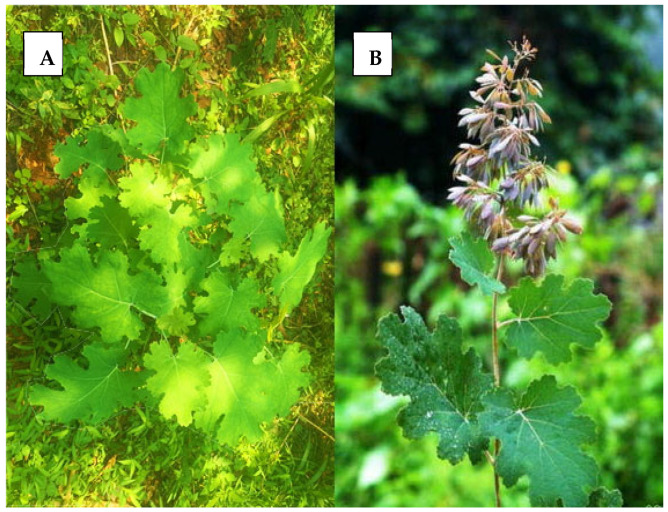
(**A**) Basal rosette of *Macleaya cordata* (plume poppy) during the vegetative stage. (**B**) Wild plant showing its aerial parts. Adapted from Lei et al. [10].

Plume poppy is a perennial herbaceous plant with a woody base that produces a milky yellow sap (Figure 1A,B). It typically grows to a height of 0.8–4 m. The plant has a thick, hollow, and erect stem that may branch in its upper parts [4]. The leaves are large, alternate, and deeply lobed, featuring a characteristic cordate (heart-shaped) base that gives the species its name. Their surfaces are typically glaucous with a waxy coating, and the margins are irregularly serrated. It produces large, conical inflorescences that can be either terminal or axillary, with a flowering and fruiting period extending from June to November [7]. Each flower lacks true petals but bears conspicuous stamens that give the inflorescence its delicate, plume-like appearance. The fruit is an elongated capsule that contains small, dark seeds. The plant is also notable for its milky orange–yellow latex, a feature characteristic of many members of the Papaveraceae family.

## 3. Sanguinarine and Chelerythrine Biosynthesis and Applications

### 3.1. Biosynthesis of Sanguinarine and Chelerythrine

**Figure 2:**
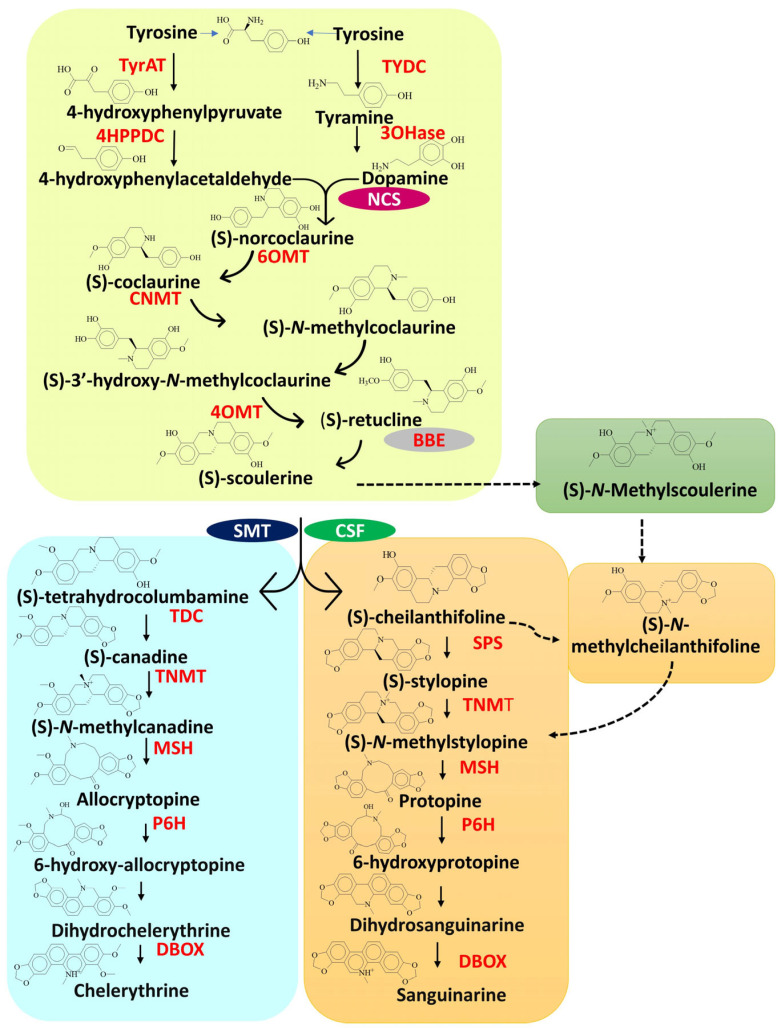
The biosynthetic pathway of sanguinarine and chelerythrine in *M. cordata* (3OHase: tyrosine 3-monooxygenase, 4HPPDC: 4-hydroxyphenylpyruvate decarboxylase, 4OMT: 3′-hydroxy-N-methylcoclaurine 4-O-methyltransferase, 6OMT: 6-O-methyltransferase, BBE: reticuline oxidase, berberine bridge enzyme; CNMT: coclaurine N-methyltransferase, DBOX: dihydrobenzophenanthridine oxidase, MSH: (S)-*cis*-N-methylstylopine 14-hydroxylase, NCS: norcoclaurine synthase, NMCH: N-methylcoclaurine hydroxylase, P6H: protopine 6-hydroxylase, SPS: stylopine synthase, TDC: (S)-canadine synthase, TNMT: tetrahydroprotoberberine-*cis*-N-methyltransferase, TYDC: tyrosine decarboxylase, TyrAt: tyrosine aminotransferase).

The biosynthesis of these BIAs involves a complex series of enzymatic reactions that begins with the condensation of two tyrosine derivatives, namely, dopamine and 4-hydroxyphenylacetaldehyde, leading to the formation of the central precursor (S)-norcoclaurine [15] (Figure 2). This reaction is catalyzed by the enzyme norcoclaurine synthase (NCS), which serves as the cornerstone of BIA biosynthesis. (S)-Norcoclaurine undergoes a series of enzymatic modifications, including methylation and hydroxylation, to form (S)-reticuline, a crucial intermediate in synthesizing many BIAs [9]. The process begins with (S)-norcoclaurine being methylated by (S)-norcoclaurine 6-O-methyltransferase (6OMT) to produce (S)-coclaurine. This is followed by the hydroxylation of (S)-coclaurine by (S)-coclaurine N-methyltransferase (CNMT), resulting in the formation of (S)-N-methylcoclaurine. Finally, (S)-N-methylcoclaurine undergoes another methylation step, catalyzed by 3′-hydroxy-N-methylcoclaurine 4′-O-methyltransferase (4′OMT), to produce (S)-reticuline. Reticuline is a key branching point in the biosynthetic pathway, as it serves as a precursor for a wide range of BIAs, including those found in plume poppy, such as protopine, allocryptopine, sanguinarine, and chelerythrine [6,9]. Once reticuline is formed, the biosynthesis splits into different pathways leading to the production of specific alkaloids. In plume poppy, these pathways lead to the synthesis of sanguinarine and chelerythrine. The formation of sanguinarine and chelerythrine begins with the oxidation of reticuline to scoulerine, catalyzed by the berberine bridge enzyme (BBE) [16]. Scoulerine then serves as a key intermediate for both sanguinarine and chelerythrine biosynthesis. Sanguinarine is formed through a series of oxidation reactions, with cytochrome P450 enzymes (protopine 6-hydroxylase, P6H; (S)-*cis*-*N*-methylstylopine 14-hydroxylase, MSH) and dihydrobenzophenanthridine oxidase (DBOX) catalyzing the final steps. In parallel, chelerythrine is produced from scoulerine via similar enzymatic oxidation steps. Understanding these pathway branches is essential for targeted metabolic engineering of specific BIAs like sanguinarine and chelerythrine.

### 3.2. Applications of Sanguinarine and Chelerythrine

The therapeutic relevance of these compounds underpin current efforts in optimizing their biosynthesis via biotechnology. BIAs have drawn significant attention due to their involvement in plant defense mechanisms, providing protection against herbivores and pathogens [4,17]. However, their significance extends far beyond their ecological roles in plants. BIAs are known for their wide array of pharmacological activities, making them crucial in the field of medicine. Some of the most well-known BIAs in plume poppy include sanguinarine and chelerythrine, which have garnered widespread recognition for their pharmacological and medicinal properties [4]. A large volume of literature is available on the applications of sanguinarine [4,6,18,19,20] and chelerythrine [4,21,22,23,24]. A brief discussion on the medicinal applications of sanguinarine and chelerythrine is presented below.

Sanguinarine, a prominent BIA, has drawn considerable attention due to its various biological activities [6]. With the growing demand for alternatives to traditional antibiotics, sanguinarine has become a key ingredient in plant-based livestock feed additives, such as Sangrovit^®^, which is approved by the EFSA [25]. However, due to the complex, planar conjugated structure of sanguinarine, its production still depends on extraction from plume poppy [9]. Sanguinarine, obtained from plume poppy, exhibits notable antimicrobial properties [26]. These characteristics enable its use as a natural growth enhancer in animal feed by inhibiting pathogenic bacteria, leading to healthier livestock and better growth outcomes [27]. The antimicrobial effects of sanguinarine can help reduce the reliance on traditional antibiotic growth promoters, which are increasingly scrutinized for their impact on antibiotic resistance [26]. Sanguinarine is also recognized for its anti-inflammatory benefits, making it a useful component in animal feed [26]. By reducing inflammation in the gastrointestinal tract, it supports gut health and enhances nutrient absorption, ultimately promoting improved growth performance [27]. This is especially beneficial for weaned piglets, as they frequently face heightened inflammation and stress during the transition to new diets. Zhao et al. [20] uncovered aspects that sanguinarine is a potential phytochemical compound for developing an effective antifungal agent. In their study, sanguinarine, along with chelerythrine and to a smaller scale, corydaline, exhibited fungicidal effects against eight species of plant pathogenic fungi, including *Fusarium oxysporum*, *Fusarium graminearum*, *Botrytis cinerea*, and *Magnaporthe oryzae*. These BIAs led to morphological abnormalities in the fungi’s mycelia, characterized by the distorted hyphae that eventually collapsed due to compromised membrane integrity. Additionally, an elevated production of reactive oxygen species (ROS) was observed in fungi treated with BIAs, particularly sanguinarine, which was linked to changes in mitochondrial redox potential and nuclear morphology [20]. Interestingly, various reports have revealed that extracts from plume poppy containing sanguinarine and other alkaloids have also been utilized as natural plant pesticides to avert bacterial and insect-related diseases in vegetable farming [28,29].

Sanguinarine also possesses strong antioxidative properties that play a crucial role in safeguarding cells from oxidative damage. This antioxidant action helps boost the immune system in animals, enhancing their overall health and resistance to diseases [30]. Research has demonstrated that adding sanguinarine to animal diets can reduce malondialdehyde (MDA) levels, a key indicator of oxidative stress, thereby improving both animal welfare and performance [26]. Moreover, numerous in vitro and in vivo studies have shown that sanguinarine exhibits anti-cancer properties across various types of cancer [19,31]. Previous studies have indicated that sanguinarine exhibits potential antitumor [32], anti-ulcer [33], antiplatelet [34], anti-angiogenesis, anti-hypertension [35], anti-osteoporosis [36], and antiparasitic properties [37]. Additionally, antibacterial mouthwashes and toothpaste containing sanguinarine were developed early in the last century [38]. However, due to concerns over its toxicity and potential carcinogenic effects, sanguinarine is currently not considered safe for clinical use and requires further research and refinement. These properties highlight its significance in both traditional and modern medicine, where further research could lead to new applications in treating various diseases. Overall sanguinarine has attracted considerable interest from both academic circles and industries because of its significant antimicrobial and growth-promoting properties in animals [8].

Chelerythrine, a benzophenanthridine alkaloid extracted from plume poppy, is valued for its wide range of pharmacological properties. It has shown promise as a therapeutic agent in the treatment of various health conditions. This alkaloid is well known for its anti-inflammatory and antibacterial properties [21,23]. Its biological activities also include anticancer [39], antifungal [40,41], and antidiabetic properties [42]. Its potential anti-inflammatory effects may involve inhibiting the production and release of exudates and prostaglandin E2 through modulation of cyclooxygenase-2 activity [21]. Both sanguinarine and chelerythrine may further contribute to anti-inflammatory responses by preventing the formation of superoxide radicals via inhibition of the phagocyte NADPH oxidase enzyme [43]. This alkaloid also helps reduce inflammation by lowering the production of inflammatory cytokines and inhibiting the NF-κB pathway, which plays a key role in the body’s inflammatory response. These properties suggest its potential use in managing chronic inflammatory conditions [22]. Studies have shown that chelerythrine extracted from plants possesses significant antibacterial properties, primarily by disrupting bacterial cell walls and membranes, which results in the leakage of cellular contents. Additionally, chelerythrine may induce oxidative stress by generating ROS and reducing the mRNA expression of essential virulence genes [44].

Chelerythrine exhibits antifungal activity, making it valuable in treating fungal infections [24,41]. The antifungal activity of plume poppy extracts containing chelerythrine reduces biofilm formation by lowering cellular surface hydrophobicity and disrupting the cAMP signaling pathway. It also enhances the cell permeability and induces ROS generation, resulting in cell death in fluconazole-resistant *Candida albicans* [24]. Furthermore, chelerythrine has been reported to exhibit anti-parasitic activity [45] and provide protection against ethanol-induced gastric ulcers [46]. Research has highlighted its potential effectiveness against cancers such as leukemia, lung cancer, prostate cancer, liver cancer, breast cancer, and melanoma, positioning it as a promising option for cancer therapy [23]. Chelerythrine has also been studied extensively for its ability to induce apoptosis in several cancer cell lines primarily through the inhibition of protein kinase C (*PKC*), a key regulator of cell growth and survival [47,48]. Similarly, chelerythrine chloride, the primary active compound in plume poppy, has been reported to induce apoptosis in human gastric cancer BGC-823 cells. This process involves a reduction in the mitochondrial membrane potential, cytochrome c release, activation of caspase-3, and cleavage of poly-ADP-ribose polymerase. It is also marked by a decrease in Bcl-xl and Bcl-2 protein levels, while Bax protein levels remain unchanged [49]. Moreover, chelerythrine has been studied for its neuroprotective properties, particularly in conditions like Parkinson’s and Alzheimer’s diseases [50]. In addition, traditional medicine has utilized extracts of plume poppy, containing chelerythrine, for wound healing purposes. Chelerythrine’s multifaceted therapeutic potential signifies its importance in modern medicine, particularly in the quest for more effective cancer treatments and alternatives to traditional antibiotics. As investigations continue, chelerythrine may play a crucial role in addressing antibiotic resistance and enhancing treatment options for various health conditions.

## 4. Transgenic Approaches for Enhancing Sanguinarine and Chelerythrine Production

BIAs in general have significant pharmaceutical potential, but the limited natural production in plants like plume poppy has hindered their commercial viability. Therefore, enhancing the biosynthesis of these compounds through transgenic methods is a potential solution. Recent advancements in biotechnology, particularly gene editing tools like CRISPR-Cas9 and metabolic engineering, have created new opportunities to boost the production of BIAs in plants. In plume poppy, these technologies can be leveraged to stimulate the expression of key enzymes involved in the BIA biosynthetic pathway, optimize metabolic flux, and improve the overall yield and stability of BIAs.

### 4.1. Gene Editing Using CRISPR/Cas9

**Figure 3:**
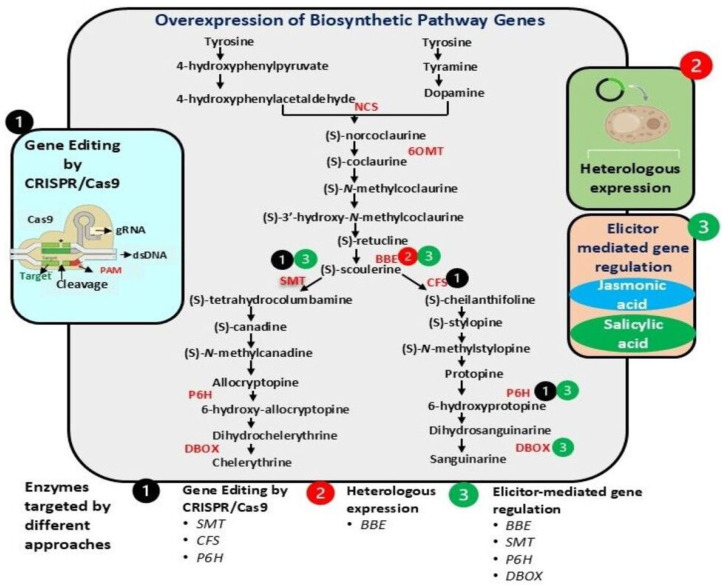
Mechanistic illustration showing potential approaches for improved sanguinarine and chelerythrine production in *M. cordata* (6OMT: 6-O-methyltransferase, BBE: reticuline oxidase, berberine bridge enzyme; CFS: cheilanthifoline synthase, DBOX: dihydrobenzophenanthridine oxidase, NCS: norcoclaurine synthase, NMCH: N-methylcoclaurine hydroxylase, P6H: protopine 6-hydroxylase, SMT: (S)-scoulerine 9-*O*-methyltransferase).

Recent successes with CRISPR in plume poppy offer potential avenues for boosting specific alkaloid yields through targeted knockouts and pathway direction [27]. CRISPR/Cas system, a versatile defense mechanism against phages found in bacteria and archaea, originates from prokaryotic organisms. This RNA-guided system enables precise genetic manipulation by recognizing DNA–RNA interactions, facilitating sequence-specific cleavage of nucleic acids [51]. Unlike previous genome-editing technologies like zinc finger nucleases (ZFNs) and transcription activator-like effector nucleases (TALENs), CRISPR/Cas9 offers several advantages, including simpler design, lower costs, and greater flexibility for creating complex or multiplex mutations [52]. Gene editing techniques such as CRISPR-Cas9 have revolutionized the field of plant biotechnology by enabling precise modifications to specific genes associated with BIA biosynthesis [2,53]. CRISPR/Cas9 has emerged as a powerful alternative to conventional breeding, enabling precise editing of biosynthetic genes in plants like *Papaver somniferum* (opium poppy) [53] and *Cannabis sativa* (hemp) [54]. Recently, using CRISPR-Cas9 gene editing technology, the optimized *BBE* gene of plume poppy were effectively incorporated into the genome of *Saccharomyces cerevisiae* (brewer’s yeast), leading to a substantial enhancement in the production of the (S)-scoulerine, key precursor of chelerythrine. The resulting yield was 58 times higher than the initial level [55]. In plume poppy, CRISPR/Cas9 technology has been specifically used to modify the genes related to sanguinarine biosynthesis. For example, editing the (S)-scoulerine 9-*O*-methyltransferase (*SMT*) gene in plume poppy led to a significant rise in sanguinarine levels, while reducing the accumulation of downstream compounds linked to the chelerythrine pathway [27]. This success highlights the potential of precise gene editing to substantially boost the production of SMs and establishes an important basis for future industrial-scale production. To enhance chelerythrine levels in plume poppy, suppression of the cheilanthifoline synthase (*CFS*) gene is necessary (Figure 3). Similarly, to increase protopine content, the expression of both *SMT* and *P6H* gene must be inhibited. CRISPR/Cas9 can be employed to introduce a targeted mutation, effectively inactivating these genes. This highlights that precise gene editing could significantly enhance the production of these alkaloids, and this targeted approach allows for more efficient accumulation of desired alkaloids while minimizing the production of unwanted side products.

### 4.2. Overexpression of Key Biosynthetic Genes

To implement metabolic engineering strategies, a fundamental understanding of the biosynthetic pathway of the target compound is essential, along with the identification of the genes responsible for encoding the key enzymes involved, especially how these genes are regulated. Numerous studies in metabolic engineering have concentrated on identifying and modifying biosynthetic genes that catalyze reactions at various stages of metabolic pathways. So far, several biosynthetic genes involved in BIA production have been identified in plume poppy [9]. Some of these genes participate in multiple branches of BIA biosynthetic pathways, including those leading to sanguinarine and chelerythrine. For instance, genes like tetrahydroprotoberberine-*cis*-N-methyltransferase (*TNMT*), *MSH*, *P6H*, and *DBOX*, are active across two branches of these pathway. Additionally, genes such as *BBE*, *CFS*, and *SMT* are present in the critical points in the biosynthetic pathways of sanguinarine and chelerythrine alkaloids. Modulating the expression of these genes, through either overexpression or silencing, has been shown to influence the accumulation of various BIAs [7,9,11]. For specific biosynthetic pathways, overexpressing genes often increases the levels of bioactive compounds. For example, overexpression of UDP-glycosyltransferase (*UGT*) genes has been linked to ROS and heightened flavonol accumulation as found in *Camellia sinensis* (tea), which in turn contributes to stress resilience and improved alkaloid content [56]. In the case of tropane alkaloids like hyoscyamine and scopolamine, gene overexpression in the tropane alkaloid pathway has been shown to directly increase the production of these medicinally valuable compounds [57].

In a recent study by Huang et al. [16], overexpression of the *BBE* gene in plume poppy resulted in significantly higher levels of (S)-norcoclaurine, (S)-coclaurine, (S)-*cis*-N-methylcoclaurine, (S)-reticuline, (S)-tetrahydrocolumbamine, (S)-tetrahydroberberine, (S)-cheilanthifoline, and (S)-scoulerine. They also found that the introduced genes in the transgenic lines showed high expression; however, levels of sanguinarine and chelerythrine were slightly reduced compared to wild-type lines, likely due to feedback inhibition caused by plume poppy *BBE* overexpression. Further Sun et al. [13] demonstrated that overexpressing the *P6H* gene in plume poppy transgenic plants significantly enhances sanguinarine and chelerythrine production with elevated expression levels of both *P6H* and the downstream enzyme *DBOX* across various plant tissues. According to Xu et al. [55], some key genes encoding rate-limiting enzymes in the BIA biosynthesis pathway in plume poppy would be potentially increase BIAs. They reported that high-yield expression of plume poppy *BBE* in yeast enhanced the effects of N-terminal modifications on enzyme performance and established a genetically stable yeast strain with elevated *BBE* expression. The regulatory gene in this plant such as *CYP719A* (a cytochrome P450 enzyme), which plays a role in the methylenedioxy bridge formation and sanguinarine synthase (SQS), which is essential for the final steps of sanguinarine and chelerythine production [9]. Some key enzymes converting intermediates into bioactive BIAs, such as DBOX and P6H, are involved in the hydroxylation steps [55]. These rate-limiting enzymes have been the focus of genetic manipulation to enhance alkaloid production in plume poppy. This overexpression of these genes could be used in producing numerous transgenic cultivars with more substantial sanguinarine and chelerythine content compared to their wild-type parents (Figure 3).

As discussed, transgenic approaches have significantly improved BIA production in plants by overexpressing key biosynthetic genes, suppressing competing pathways, and introducing novel metabolic genes to increase BIA yield. Efforts include overexpressing rate-limiting enzymes and using techniques like gene knockout or silencing to redirect metabolic flux toward alkaloid production, enhancing yields in plume poppy (Table 1). Gene overexpression has shown significant advancements in improving BIA production in other plants. Overexpressing genes involved in BIA biosynthesis pathways, such as *NCS* and *6OMT*, has been particularly effective [58]. For instance, the increased production of morphine, sanguinarine, and other key alkaloids have achieved by overexpression of these enzymes in opium poppy [59]. These findings contribute to the metabolic engineering field by establishing foundational methods for efficient BIA biosynthesis and guiding further optimization efforts for complex biosynthetic pathways.

By optimizing the expression of these enzymes, researchers can channel more resources into BIA production. Additionally, metabolic engineering allows for the introduction of synthetic pathways or the incorporation of genes from other BIA-producing species, further enhancing alkaloid yields. Some strategies that would be beneficial for optimizing the expression of overexpressed gene for BIAs involve, first, fine tuning gene regulation by promoter optimization. Use of tissue-specific, strong, or inducible promoters can pointedly enhance the expression levels of overexpressed genes such as extensive use of CaMV 35S promoter in plant species to drive the expression of BIA pathway genes like *NCS* and *6OMT* [62]. The second strategy would involve adapting codons of the overexpressed genes to the host plants preferred codon usage to improve translation efficiency. It has been applied in transgenic opium poppy and plume poppy and achieved higher BIA content by optimizing codons for enzymes CYP719A and BBE [7]. The third strategy would be targeting specific enzymes to the subcellular compartments such as vacuoles. Plastids where certain substrates accumulate can enhance the efficiency of BIA biosynthesis [62]. Finally, the fourth strategy would be pathway balancing by avoid metabolic bottlenecks. Simultaneous multi-gene overexpression from different steps of the BIA biosynthesis pathway can augment flux. For illustration, overexpressing both *NCS* and *CNMT* in *Papaver* species led to the amplified production of intermediates and final alkaloid products [62]. This strategy is helpful to prevent accumulation of unwanted metabolites by supplying balanced precursors and intermediates. In addition to these strategies, recent studies have directed that expression of BIA biosynthesis genes to the chloroplasts rather than the nucleus can significantly increase alkaloid yields due to higher gene copy numbers and transcriptional activity in chloroplasts [7]. Fine tuning these targets using overexpression has already shown success in increasing precursor and final product accumulation in transgenic lines.

### 4.3. Enhancing BIAs, Alkaloid Delivery, and Stability

Alkaloid accumulation is not only about synthesis. Their transport and storage also determine final yield and efficacy. One of the major challenges in harnessing the therapeutic potential of BIAs is ensuring their stability and bioavailability. Alkaloid stability in plants is often affected by ecological factors such as temperature and light [63]. Moreover, plant–cell chemical interactions affect the alkaloid stability through the plant matrix, pH, and presence of other phytochemicals [64]. Generally, alkaloids have low water solubility, and their interactions with fibers, proteins, and fats in plants can influence their release, absorption, and metabolism, which in turn impact the bioavailability of the alkaloid [65,66]. The stability and bioavailability of alkaloids would be enhanced by encapsulation techniques. More investigations should be performed to assess the synergy between alkaloids and other plant constituents; for example, piperine increased the bioavailability of some alkaloids in *Piper nigrum* (black pepper) [67,68].

Transgenic approaches can be used to improve the intracellular storage and transport of BIAs, thus reducing their degradation and improving their therapeutic efficacy. BIAs have complex biosynthetic pathways, and optimizing their transport and accumulation in specific plant tissues can greatly enhance yields. Transport of these alkaloids involves membrane-bound transporter proteins, especially ATP-binding cassette (ABC) and multidrug and toxic compound extrusion (MATE) transporters [68]. BIA transporters facilitate their movement across membranes, and their genetic tunning has been a successful approach in increasing BIA transport and subcellular compartmentation [69,70]. Other than exploring the use of molecular transporters, some studies have explained vacuole-targeting strategies to sequester BIAs within plant cells, protecting them from enzymatic degradation [71,72]. Moreover, targeted transgenic approaches, such as the use of tissue-specific promotors for easier harvesting parts of plants, like roots, leaves, or flowers, enables selective transport and storage of BIAs [73,74].

In addition to enhancing sanguinarine and chelerythine biosynthesis, transgenic approaches can be used to improve the transport and storage of alkaloids within the plant. Overexpressing transporter proteins that facilitate the movement of BIAs from the site of synthesis to storage compartments could increase the overall alkaloid content. Furthermore, engineering plants with enhanced vacuole storage capacity may help to sequester toxic alkaloids, thereby protecting the plant and enabling higher production levels. Additionally, elicitation has recently gained attention as a valuable biotechnological strategy to elevate the production of SMs in crops. Elicitor compounds, derived from both abiotic and biotic sources, trigger plant defense responses and enhance secondary metabolic pathways. This process helps the plants maintain both productivity and resilience [75]. Strategies combining gene overexpression with elicitors like jasmonic and salicylic acids have been proven effective in boosting alkaloid biosynthesis. Such approaches also demonstrate potential in commercial applications, especially with advances in bioreactor systems and nanotechnology to further amplify these effects [57,59]. Recently, Huang et al. [60] reported that different elicitors, especially methyl jasmonate, boost sanguinarine and chelerythine production in plume poppy. Among the tested treatments, methyl jasmonate alone proved most effective, increasing sanguinarine and chelerythine levels by 10- and 14-fold, respectively, and upregulating key biosynthetic genes. In addition, some studies on other plants, such as *Coptis* species and opium poppy have shown that that genetic modifications along with elicitors such as methyl jasmonate can upregulate the expression of BIA biosynthesis [76,77]. These strategies enhance both the accumulation and stability of BIAs, making them more suitable for pharmaceutical applications.

### 4.4. Metabolic Engineering and Pathway Diversification

Beyond boosting native pathways, synthetic biology enables the transfer of entire BIA biosynthetic routes into chassis organisms. In plants, metabolic engineering aims to boost the production of various classes of BIAs such as sanguinarine, chelerythine, protopines, and berberine. As discussed, the foundation for this engineering starts with pathway optimization and gene overexpression of rate-limiting steps in BIA pathways [74,78]. Another important step in this engineering would be to introduce heterologous pathways by entering BIA biosynthetic pathways into model plants or other higher-biomass plants for traditional BIA production [73,79]. Ensuring ample precursor availability (e.g., tyrosine, dopamine) is essential to avoid upstream bottlenecks in engineered pathways [2]. In metabolic engineering, it is worth considering that BIAs are often toxic at high levels, so compartmentalization of BIA pathways should be carried out within plant organelles [80]. The final step for this engineering is the regulation of the biosynthetic pathways by fine tuning of the regulatory proteins of MYB and bHLH transcription factors. This is possible with targeted gene modifications using CRISPR technology [81,82]. However, there is a need for further developments to identify bottlenecks and regulatory points within BIA pathways, which can be achieved using advanced metabolomics, transcriptomics, and proteomics approaches [83]. Further, the integration of system biology tools with metabolic engineering can help researchers predict the outcomes of genetic modifications that could be more effective for BIA production [80]. Together, these tools allow for both production enhancement and structural diversification, leading to novel or improved BIAs.

To expand the range of alkaloids to create novel compounds in plants demands pathway diversification, particularly BIA production pathway. The development of hybrid pathways by incorporating enzymes of different BIA pathways that could be from plants or even microbes is important to demonstrate a combinatorial biosynthesis within a single host for novel alkaloids [84,85]. The introduction of cross-kingdom biosynthetic pathways by integrating designed microbial biosynthetic genes into plant genomes not only diversifies BIA products but also would be beneficial for amplify the alkaloid production in plants [86,87]. Some other efforts for diversification would be engineering of promoters or transcription factors for regulating pathway branching [88,89], bypass of traditional pathways with the use of synthetic enzyme cascades [90], and the use of targeted enzymes for subcellular compartmentalization [91]. However, there is a need of higher throughput studies for identification of new pathway enzymes and intermediates, revealing unexplored branches of BIA biosynthesis.

## 5. Conclusions and Future Prospects

The current review highlights the potential application of gene editing and metabolic engineering in significantly improving sanguinarine and chelerythrine in plume poppy. It also underscores the identification and manipulation of key biosynthetic pathways, optimization of metabolic flux, and utilization of elicitors to enhance BIA production. The present review also validates the role of transgenic strategies over traditional approaches, which limit the metabolic yields. However, fine mapping of transcription factors and post translational modifications are necessary for fully optimizing their biosynthetic pathways. Conclusively, risk assessments and gene containment strategies on transgenic medicinal plants are crucial for long-term success. Therefore, a more critical reflection on scalability, commercial feasibility, and clinical translation will enrich future experiments and investments in the field of plant-based pharmaceutical biotechnology.

## Figures and Tables

**Table 1:** The key regulatory gene enzymes targeted using different approaches for enhancement of specialized metabolites.

Target Gene/Purpose in *Macleaya cordata* (Plume Poppy)	Gene Manipulation Technique	Specialized Metabolites	References
Knockout of *SMT*	CRISPR/Cas9 mediated disruption of chelerythrine biosynthesis	Elevated sanguinarine levels by 3.29-fold	[27]
Upregulated expression of *BBE*	Endogenous expression of plume poppy *BBE*, a crucial enzyme for converting (S)-reticuline to (S)-scoulerine	Sanguinarine and chelerythrine were slightly reduced due to feedback inhibition of plume poppy *BBE* overexpression	[16]
Overexpression of *BBE*	Optimization and heterologous expression of a gene of plume poppy *BBE*	Elevated (S)-scoulerine, key precursor of chelerythrine yield by 58 times higher than the original level	[55]
Upregulated expression of *P6H* and *DBOX*	Elicitor-mediated gene regulation	Elevated sanguinarine and chelerythrine content by 10 and 14-fold, respectively	[60]
Upregulated expression of *P6H*	Over expression of plume poppy *P6H*	Elevated sanguinarine and chelerythrine production	[13]
Upregulated expression of *P6H* and *DBOX*	Induction of hairy roots	Elevated sanguinarine production	[61]

Abbreviations: BBE: reticuline oxidase, berberine bridge enzyme; DBOX: dihydrobenzophenanthridine oxidase, P6H: protopine 6-hydroxylase, SMT: (S)-scoulerine 9-*O*-methyltransferase.
